# Microbial Modulation of the Gut–Liver Axis in Autoimmune Liver Diseases

**DOI:** 10.1055/a-2679-3641

**Published:** 2025-08-27

**Authors:** Shihui Wei, Juan Lu

**Affiliations:** 1 State Key Laboratory for the Diagnosis and Treatment of Infectious Diseases, National Clinical Research Center for Infectious Diseases, National Medical Center for Infectious Diseases, Collaborative Innovation Center for Diagnosis and Treatment of Infectious Diseases, The First Affiliated Hospital, Zhejiang University School of Medicine, Hangzhou City, China

**Keywords:** autoimmune liver diseases, gut–liver axis, gut microbiota, intestinal dysbiosis, microbial metabolites, microbial biomarkers

## Abstract

Autoimmune liver diseases (AILDs), including autoimmune hepatitis, primary biliary cholangitis, and primary sclerosing cholangitis, are chronic inflammatory conditions influenced by complex interactions among genetic, environmental, and immunological factors. Recent studies have highlighted the critical role of the gut microbiota in regulating immune responses beyond the gastrointestinal tract via the gut–liver axis. This review examines the interactions between intestinal microecology and AILDs, with a focus on mechanisms such as bacterial translocation, disruption of the intestinal barrier, and modulation of microbial metabolites. Dysbiosis, involving alterations in both bacterial and fungal communities, has been associated with immune dysregulation and hepatic inflammation. Evidence indicates that short-chain fatty acids, bile acids, and microbial products such as lipopolysaccharides influence hepatic immune tolerance and inflammatory signaling pathways. Several diagnostic and therapeutic approaches, including probiotics, fecal microbiota transplantation, and bile acid regulation, have shown potential to slow or alter disease progression. However, the clinical translation of these findings remains limited due to interindividual variability and the complex nature of the gut–liver axis. Continued research is needed to develop precision medicine strategies that can harness intestinal microecology for improved management of AILDs.

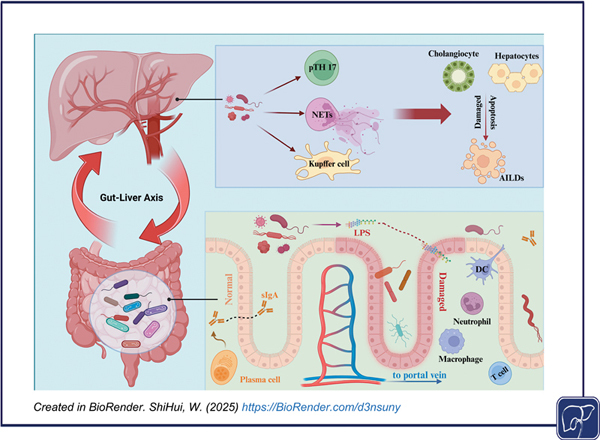

## Basic Characteristics of Gut Microbiota

The gut microbiota represents a highly complex ecosystem composed of a diverse range of microorganisms, including bacteria, archaea, viruses, eukaryotes, and fungi ( Table 1 ). Genomic analyses have revealed that the collective gene pool of the gut microbiome far exceeds that of the human genome, earning it the designation of the “second human genome.” Notably, more than 99% of the genes within the human gut microbiota are derived from bacteria. 3 Although fungal biomes represent less than 1% of the total human gut microbiota, 4 and the Human Microbiome Project has indicated that the diversity of fungi in the human intestine is considerably lower than that of bacteria, with notable inter- and intraindividual variations, 5 numerous early investigations have illustrated the role of fungi in disease development and their significant influence on the host's immune system. 6 7 Wu et al 8 suggested core microbiome characteristics that can serve as indicators of health. By examining metagenomic data from a high-fiber dietary intervention aimed at type 2 diabetes, along with 26 case–control studies across 15 diseases, they discovered genome pairs consistently linked in coabundance networks during dietary interventions and disease disruptions. These genomes formed a framework of “two competitive gut communities” that effectively distinguished cases from controls for various diseases and forecasted immunotherapy results. The key functional group (C1A) comprises solely species from the phylum Firmicutes, which are specialized in fermenting fiber and producing butyrate, thereby benefitting host health. Members of C1A are rich in carbohydrate-active enzyme (CAZy) genes, enabling them to break down complex plant polysaccharides, as well as in genes linked to butyrate synthesis, including the but gene. Supporting this notion, Lau et al further illustrated how specific microbial alterations and metabolites—including short-chain fatty acids (SCFAs)—affect immune responses and shape cancer immunotherapy outcomes. 9

**Table 1:** The primary constituents of the intestinal microbiota

Category	Species	Effection	References
Bacteria Fungi	*Firmicutes* *Bacteroidetes* *Actinobacteria* *Proteobacteria* *Fusobacteria* *Verrucomicrobia* *Candida* *Saccharomyces* *Penicillium* *Aspergillus* *Cryptococcus* *Malassezia* *Cladosporium* *Galactomyces* *Debaryomyces* *Trichosporon*	The *Lactobacillus* and *Clostridium* are included, as they have been reported to assist with the digestion of complex carbohydrates, the fermentation of simple sugars, and the metabolism of short-chain fatty acids. These bacteria are frequently observed in greater abundance in obese individuals. The *Bacteroides* spp. is of particular significance within the colon, occupying an important position and was observed predominantly in adults like the Firmicutes. The Bifidobacterium is the primary focus, especially *B. longum* , is capable of utilizing oligosaccharides present in breast milk as a carbon source, which might inhibit the growth of other bacteria which enables it to flourish in the infant gut. A small proportion of a healthy intestine, but its diversity and ability to respond to environmental changes make it unique in the intestinal microecology. An increase in the abundance of it is considered to be one of the characteristics of intestinal flora imbalance. Fusobacteria account for a small proportion of healthy intestines, but studies have found that their abundance increases significantly in some pathological conditions, such as IBD and colorectal cancer. The primary representative is *Akkermansia muciniphila* , a bacterium that inhabits the mucus layer of the intestine, breaks down mucin glycoproteins, encourages the production of mucus, and supports the intestinal barrier's integrity. Furthermore, it aids in the production of SCFAs and vitamin B12. Ten core species of intestinal fungal pathogens have been identified. They are capable of synthesizing vitamins B and vitamin D, which affects and shapes the host's immune system. Furthermore, the body is capable of mounting an immune response against pathogens and developing tolerance to beneficial bacteria by activating fungal-specific pathogen recognition receptors (PRRs).	115 116 117 118 119 120 121 122 123 124 125 126 127
Virus	Bacteriophage	Phages play a role in regulating the structure of bacterial communities and promoting horizontal gene transfer in bacterial populations.	128
Archaeon	*Methanogenic archaea*	*Methanogenica smithia* is closely related to adults and plays an important role in regulating the host's energy balance.	129 130

Notes: This table summarizes the microbial species and the general functions they perform as described in numerous peer-reviewed research and review articles on the healthy human gut microbiota. Due to the variations in the designs of the original studies including the use of clinical cohorts, animal models, and in vitro studies, we do not have detailed information on sample size, subject characteristics and type of studies. As a result, this table presents a broad overview of commonly reported taxa and their functions, not a quantitative meta-analysis.

## Mechanisms Linking Intestinal Microecology to Autoimmune Liver Diseases

The detrimental impacts of intestinal dysbiosis on the health of the host have been recognized for a significant duration. Evidence has shown that intestinal dysbiosis can provoke autoimmune reactions via several mechanisms, such as helper T cell bias, paracrine activation, antigen-determining site spreading, cross-reactivity, and the recognition of microbial antigens by dual T cell receptors. 10 Recent studies have indicated that the gut microbiota, as a key environmental factor, is crucial in the development of AILDs. Presently, it is believed that intestinal dysbiosis may lead to the onset of AILDs by activating specific signaling pathways, modifying gut microbiota metabolism, and affecting the regulation of the intestinal barrier, among other factors.

### Intestinal Barrier Function and Microbial Translocation

#### Intestinal Barrier Function

**Fig. 1:**
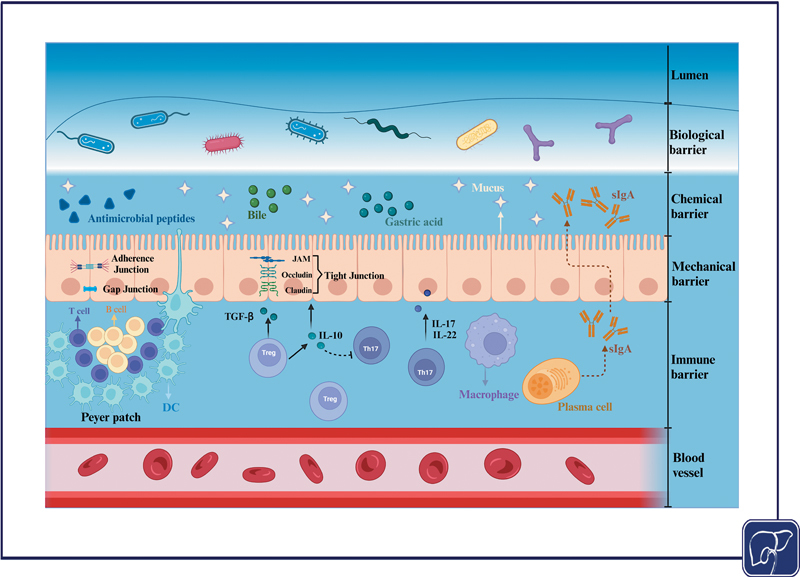
The multilayered intestinal barrier: a collaborative defense system maintaining homeostasis. *Created in BioRender. ShiHui, W. (2025)* *https://BioRender.com/u73b050* .

Bacterial translocation, which refers to the movement of viable bacteria or their products from the intestinal lumen to mesenteric lymph nodes or other extra-intestinal organs, has been shown to be commonly linked with hepatobiliary diseases. It is believed that dysfunction of the intestinal barrier serves as the underlying pathological basis for this occurrence. Preserving the integrity of the gut barrier is essential to stop the microbiota from triggering an adaptive immune response in healthy individuals. This barrier consists of four primary components that occur in succession: biological, chemical, mechanical, and immunological ( Fig. 1 ). Research has indicated that the liver, particularly the Kupffer cells, functions as an “intravascular immune firewall” (via the gut–liver axis of microbial D-lactate) to restrict the further dissemination of enteric pathogens by capturing and eliminating bacteria during instances of increased intestinal permeability, a condition commonly referred to as “leaky gut syndrome.” 11 However, this can also render hepatocytes susceptible to immune stimulation from gut microbiota and their metabolites. The inflammasome pathway is currently recognized as a key molecular mechanism for sustaining epithelial integrity and intestinal homeostasis. Among these pathways, the NLRP3 inflammasome enhances inflammation through caspase-1-dependent activation, which involves the cleavage of pro-inflammatory cytokines interleukin (IL)-1β and IL-18, in addition to inducing pyroptotic cell death. 12 Prior studies have indicated that a leaky gut resulting from NLRP3 deficiency can lead to elevated levels of toll-like receptor 4 (TLR4) and TLR9 agonists (such as lipopolysaccharides [LPS]) within the portal vein, consequently prompting hepatocytes to generate tumor necrosis factor (TNF) and advancing the progression of liver diseases. 13 Moreover, scholars propose that additional mechanisms could also contribute to the development of a leaky gut. The currently investigated mechanisms include physical trauma, toxins, disruption of tight junctions (TJs), alterations in epithelial stem cell turnover, and modifications in the consistency of the mucus layer. 14 15

The intestinal barrier comprises several layers of defense mechanisms—biological, chemical, mechanical, and immune—that collaborate to sustain intestinal homeostasis. The biological component includes gut microbiota, which is essential for shielding the host from pathogens. Meanwhile, the chemical aspect features mucus, antimicrobial peptides, and digestive secretions like bile and gastric acid, which deter the attachment and invasion of pathogens. The mechanical barrier is established by tightly linked intestinal epithelial cells, bolstered by proteins such as occludin and claudin, which uphold the integrity of the intestinal mucosa. The immune layer is characterized by gut-associated lymphoid tissue, which includes Peyer's patches, where Treg cells and Th17 cells play a role in managing immune responses. Treg cells release TGF-β and IL-10 to foster immune tolerance and curb excessive inflammation, whereas Th17 cells generate IL-17 and IL-22 to boost mucosal immunity. Additionally, plasma cells within the intestinal immune barrier produce sIgA, aiding in the establishment of the chemical barrier. Immune cells, including macrophages, further modulate the immune response in the gastrointestinal tract ( Fig. 1 ).

#### Microbial Translocation: Gateways to Liver Inflammation through the Gut Barrier

Damage to the intestinal barrier enhances the dynamic translocation and modification of gut microbiota, thereby affecting the development and progression of liver diseases. Nakamoto and colleagues found that *Klebsiella pneumoniae* is present in the gut microbiota of patients with PSC. 16 They showed in a mouse model that *K. pneumoniae* prompts human intestinal epithelial cells to create pores, resulting in the disruption of the intestinal epithelial barrier and triggering dysbiosis. After introducing the microbiota from PSC patients into germ-free (GF) mice, *K. pneumoniae* , *Proteus mirabilis* , and *Enterococcus faecium* were successfully cultured and isolated from the mesenteric lymph nodes of these mice. Together, these three bacterial species contribute to the advancement of hepatobiliary disorders through the TH17 immune response. Importantly, *K.* *pneumoniae* is pivotal in mediating the impact of intestinal epithelial damage on the activation of the liver's TH17 response.

In recent years, the issue of liver damage due to fungal translocation has received considerable attention. β-glucans, the primary components of fungal cell wall polysaccharides, is a critical pathogen-associated molecular pattern that interacts with various pathogen recognition receptors in the body, including Dectin-1, complement receptor 3, and TLR4, which are crucial for initiating innate immune responses. 17 18 Yang et al 19 have shown that when the intestinal barrier is compromised, β-glucans, particularly those produced by *Candida parapsilosis* , can enter the liver via the bloodstream. In the liver, β-glucans interacts with the dectin-1 on Kupffer cells, leading to an increase in the expression and secretion of IL-1β, ultimately causing hepatocyte damage in mice. However, *Candida albicans* is the predominant species in the human gut mycobiome, rather than *C. parapsilosis* . Therefore, the compositional changes identified in the article may not substantiate a causal relationship with liver disease progression. Additionally, the structural differences in β-glucans derived from various fungi could influence the immunogenicity of β-glucans, thereby exhibiting different effects on the liver. Although a direct causal link between β-glucans and AILDs remains to be established, existing mechanistic evidence suggests a potentially relevant immunological axis: β-glucans binding to dectin-1 on Kupffer cells activates the Syk/NF-κB signaling cascade, leading to IL-1β and IL-6 production—cytokines known to promote Th17 cell differentiation. This is particularly relevant in AIH and PSC, where aberrant Th17 responses have been implicated in pathogenesis.

Research has indicated that additional fungal metabolites may also be transferred to the liver through a damaged intestinal barrier. Candidalysin, a peptide toxin secreted by *C. albicans* , may directly induce hepatocyte death through various mechanisms, including the activation of the MAPK/c-Fos/MAP kinase phosphatase 1 signaling pathway. 20 Moreover, specific fungi located in the intestinal lining can transmit signals to the liver through the movement of antigen-reactive cells, including Th17 cells, which could also trigger abnormal responses in the liver. 21 Evidence suggests that patients with PSC and choledochal candidiasis experience more severe cholangitis, accompanied by elevated C-reactive protein and serum bilirubin levels, whereas patients without candidiasis do not experience these conditions. 22

Therefore, while the contribution of fungi to the pathogenesis of AILDs remains hypothetical, these findings collectively support a conceptual framework wherein fungal components—by activating innate immune receptors and modulating the Th17 axis—may amplify hepatic autoimmunity in genetically susceptible hosts. Further studies are warranted to validate this hypothesis in autoimmune settings.

### Within-Host Evolution of Gut Pathobionts

Numerous research efforts indicate that distinct strains of microorganisms can adapt and evolve throughout an individual's life span as a response to the evolution of their host. Similar to other populations, the evolutionary processes affecting intestinal microbes predominantly involve migration, mutation, genetic drift, natural selection, and recombination. Each day, the gut microbiota generates billions of new mutations, contributing to the diversification and adaptation of symbiotic microbes. Importantly, findings from mutation accumulation experiments suggest that the majority of these mutations are detrimental. Current understanding posits that horizontal gene transfer (HGT), clonal interference, and selective sweeps significantly influence the mutation process; however, the exact mechanisms remain inadequately comprehended. 23

Yi et al 24 performed a series of in vivo experimental evolution studies alongside comparative genomics research in mice, demonstrating that the intestinal pathogen *Enterococcus gallinarum* evolved into distinct strains, each with different ecological niches and levels of pathogenicity due to host-driven evolution. One particular strain has adapted to thrive in the intestinal lumen and is highly vulnerable to immune responses, whereas another strain is better equipped for mucosa colonization and possesses the ability to evade immune detection and clearance. Additionally, the strain adapted to the mucosa shows an increased capacity for transepithelial transport, which aids its movement to the mesenteric lymph nodes and liver. This strain exhibits a higher rate of metastasis and survival in these sites, leading to more severe inflammation in the intestines and liver. In contrast to typical pathogenic bacteria, which the immune system can quickly eliminate, these bacteria tend to remain somewhat concealed within organs for a brief duration, managing to avoid detection by the immune system. Nonetheless, their prolonged presence may result in pathological consequences, such as the onset of autoimmune diseases. This situation offers some insight into why certain individuals harboring potentially harmful bacteria do not develop illnesses, even as their risk for disease escalates with aging. Beyond *E. gallinarum* , similar situations might also be observed with other intestinal pathogens. Yaffe and Relman 25 established a technique that utilizes high-throughput chromosome conformation capture along with a probabilistic noise model to monitor evolution. Leveraging advanced technologies to investigate the evolution and adaptive transformations of other potential pathogens, along with their interactions with the host immune system, will enhance our understanding of the gut microbiota's influence on the host's autoimmune disorders.

### Gut Microbial Metabolites

A growing body of evidence indicates a strong correlation between alterations in the metabolites produced by the gut microbiota and the development of various immune-related inflammatory diseases. To date, only a restricted variety of microbial metabolites have been identified; however, these metabolites are highly diverse and their functions have been the subject of extensive investigation ( Table 2 ). These metabolites may affect the functioning of various immune cells, such as T cells, B cells, dendritic cells (DCs), macrophages, among others, implicating them in the development of immune-mediated inflammatory diseases.

**Table 2:** Gut flora metabolites and their main effects

Source	Metabolites	Receptors	Functions
Dietary components	SCFAs	GRP41 GRP43 GRP109a	Promote T cells differentiation 131 132 Regulate cytokines production (e.gIL-10,IL-22) 34 35 133 Promote B cells activation and antibody secretion 134 Regulate Neutrophil activity and function 135 Stimulate dendrite elongation in DCs 136 inhibit the activation of BMDCs via suppressing the LPS-mediated expression of co-stimulatory molecules and the production of cytokines 137 ; Affect macrophage activity and metabolism, regulate the polarization of macrophages 138
Produced by the host and modified by gut bacteria Synthesized de novo by gut microbiota	Secondary bile acids LPS BACCs	FXR TGR5 TLR4 mTOR	A metabolic homeostat for bile acid, glucose and lipid metabolism in the liver 40 Inhibit the differentiation of TH17 cells, while enhance the differentiation of Treg cells 139 Modulate NLRP3 Inflammasome activation to regulate macrophage inflammation 140 Promote ISC renewal and drive regeneration in response to injury 141 Control the accumulation of CXCR6 + NKT cells in the liver by regulating the level of CXCL16 on LSEC 142 Stimulates the formation of NETs 59 Induce the secretion of inflammatory factors (e.g., TNF-α,IL-6, IL-1β) and the production of GM-CSF 51 Downregulates the expression of the TGF-β pseudo-receptor Bambi 143 Promote anabolism(e.g., protein translation) and inhibit catabolism(e.g.autophagy) 144 Stimulate peripheral blood mononuclear cells, resulting in a significant increase in IFN-γ production 145 Synergistically reducing the number of CSCs and enhancing HCC chemotherapy sensitivity 146

Abbreviations: BACCs, branched-chain amino acids; BMDCs, bone-marrow-derived DCs; CAR, constitutive androstane receptor; CSCs, cancer stem cells; FXR, farnesoid X receptor; GM-CSF, granulocyte–macrophage colony-stimulating factor; HCC, hepatocellular carcinoma; IFN-γ, interferon-γ; ILCs, innate lymphoid cells; ISC, intestinal stem cell; LSEC, liver sinusoidal endothelial cells; NETs, neutrophil extracellular traps; PXR, pregnane X receptor; TGR5, Takeda G protein-coupled receptor 5; VDR, vitamin D receptor.

#### Short-Chain Fatty Acids

**Fig. 2:**
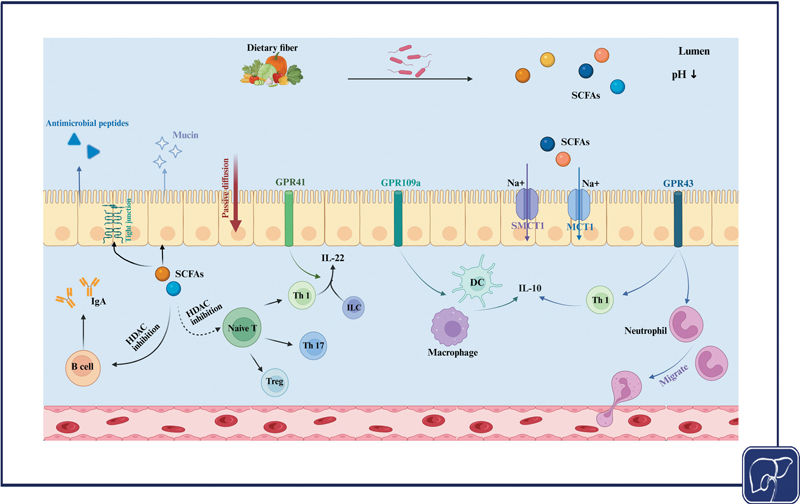
Cellular entry and immunoregulatory mechanisms of short-chain fatty acids. *Created in BioRender. ShiHui, W. (2025)* *https://BioRender.com/i21t449* .

Carbohydrates that are not fully digested may undergo fermentation by gut microbiota, particularly by species such as *Faecalibacterium prausnitzii* , *Blautia* , *Bifidobacterium* , and *Bacteroidetes* , leading to the production of SCFAs ( Fig. 2 ). Various SCFAs are distributed in distinct regions of the intestine: acetate and propionate can be found in both the small and large intestines, whereas butyrate is predominantly located in the colon and cecum. SCFAs play several roles, such as fostering the production of antimicrobial substances, regulating the turnover of epithelial cells, and preserving the integrity of the epithelial barrier. For instance, butyrate enhances the expression of TJs and stabilizes the levels of hypoxia-inducible factor, thereby strengthening the intestinal barrier. 26 27 Additionally, SCFAs influence a variety of immune cells—including T cells, B cells, and macrophages—via the G protein-coupled receptors (GPCRs) signaling pathway. 28 Furthermore, SCFAs provide a protective effect in the intestine by hindering the establishment of pathogenic bacteria, such as *C. albicans* , through the acidification of the gut environment. 29 These fatty acids also exhibit immunomodulatory effects on organs outside the intestine. Among them, butyrate is particularly influential in the gut–liver axis, where it can trigger the production of IL-18 by Kupffer cells and hepatocytes in a manner dependent on the GPR109A receptor, and IL-18 has been shown to be linked with mitochondrial function and the maturation of natural killer cells in the liver. 30 The proliferation and growth of liver cells are reliant on the biosynthesis of membrane phospholipids, and the metabolites from gut microbiota travel through the gut–liver axis, significantly aiding in lipid biosynthesis.

Yin et al 31 therefore investigated the role of gut microbiota in promoting liver regeneration through the biosynthesis of hepatic membrane phospholipids, with a particular focus on the link between SCFAs in gut microbiota and liver lipid metabolism. They used C57Bl/6J wild-type mice to perform a 70% partial hepatectomy (PHx) experiment, with some mice treated with broad-spectrum antibiotics before surgery to induce intestinal dysbosis, while using GF mice and oligo-mouse-microbiota (OMM) for comparison. It was found that the antibiotic-treated mice showed a decrease in α diversity of the gut microbiota, an increase in Proteobacteria in particular, and a decrease in SCFAs-producing flora, accompanied by a decrease in the expression of stearoyl-CoA desaturase 1 (SCD1), which catalyzes the formation of monounsaturated fatty acids. SCD1 was found to be SCFAs-inducibly expressed in vitro and in vivo and is necessary for human liver cancer cell proliferation. In addition, SCD1 was also found to be associated with liver regeneration in human patient tissue biopsies. These mice had delayed liver regeneration and impaired hepatocyte proliferation after hepatectomy. In contrast, GF mice had impaired liver regeneration after hepatectomy, whereas the OMM partially recovered their liver regeneration capacity. This study for the first time revealed that SCFAs have the function of promoting liver regeneration, providing new ideas for further research on the treatment of liver disease with the gut microbiota.

SCFAs can enter cells through three main pathways: simple diffusion, carrier-mediated transport involving the transporters SMCT1 (SLC5A8) and MCT1 (SLC16A1), and activation of GPCRs. These SCFAs have the ability to modulate immune cell functions by inhibiting histone deacetylase (HDAC) and/or activating GPRs such as GPR41, GPR43, and GPR109A. 32 33 Both GPR41 and GPR43 interact with acetate, propionate, and butyrate, whereas GPR109A is primarily activated by butyrate. SCFAs can influence the differentiation of naive T cells and suppress the release of proinflammatory cytokines through HDAC inhibition, potentially prompting intestinal B cells to generate IgA. The stimulation of GPR41 has been shown to promote the production of IL-22 by CD4+ T cells and innate lymphoid cells. In contrast, the activation of GPR43 leads to the production of IL-10 by Th1 cells and alters the migration and activities of neutrophils during inflammatory responses. 34 35 36 Regarding GPR109A activation, research indicates that it inhibits cyclic adenosine monophosphate production and encourages colonic DCs and macrophages to release IL-10 Fig.2 37

#### Secondary Bile Acids

Primary bile acids (BAs) are synthesized primarily in the liver and subsequently excreted into the intestines. Intestinal bacteria facilitate the conversion of primary BAs into secondary BAs, including deoxycholic acid (DCA) and lithocholic acid (LCA), through a series of enzymatic processes, including dehydrogenation, 7α-dehydroxylation, and exoisomerisation. As well as promoting digestion and having an antibacterial effect, secondary BAs also play an important role in immune regulation. 38 To date, the BA receptors that are well documented and closely associated with hepatobiliary diseases are primarily farnesoid X receptor (FXR) and TGR5.

FXR serves as a transcription factor whose activation is crucial for restoring homeostasis in both the intestinal and gut vascular barriers. 39 Furthermore, it is believed that processes such as energy regulation, autophagy, inflammatory responses, and fibrosis are significantly associated with FXR. 40 Similarly, it regulates the transcription of key BA synthesis enzyme genes Cyp7a1 and Cyp8b1 through the endocrine pathway of fibroblast growth factor 15/19 in the ileum, in response to postprandial or abnormal gut–liver BA flow. 41 Leveraging these functions, researchers have found that numerous medications can mitigate different models of liver damage through the activation of the FXR signaling pathway. 42 43 44 In recent years, there has been a growing interest in the use of FXR agonists for the treatment of PBC. Initial investigations indicated that LCA can enhance the levels of apoptosis-related proteins, including cleaved poly (ADP-ribose) polymerase (PARP) and cleaved caspase-3, leading to hepatocyte apoptosis in murine models. 45 Building on this, Lu et al 46 noted that in mice with cholestatic liver injury induced by LCA and pretreated with obeticholic acid (OCA), OCA not only alleviated cholestasis by downregulating BA export transporters but also reduced LCA-induced hepatocyte apoptosis by lowering the levels of apoptosis-related proteins such as cleaved caspase-3, cleaved caspase-8, and cleaved PARP. This finding offers fresh insight into the role of FXR agonists in combating cholestatic liver injury. In addition, Tang et al innovatively revealed using a mouse model that hepatocyte FGF4 might be a direct downstream target of hepatic FXR, which signals to transcriptionally control Cyp7a1 and Cyp8b1 expression through a heretofore uncharacterized intracellular FGFR4-LRH-1 signaling node within the liver. 47 However, these findings necessitate further validation to determine whether the beneficial effects of FGF4 and related pathways on BA metabolism observed in mice can be replicated in humans and their safety.

TGR5 functions as a receptor for bile salts that is coupled to G-proteins. Initial research indicated that TGR5 not only induces nitric oxide production in rat liver endothelial cells and decreases the cytokine gene induction in LPS-activated rat Kupffer cells by inhibiting the NF-κB signaling pathway, thus producing anti-inflammatory effects, but it also safeguards the liver from BAs overload consequences following PHx by regulating bile hydrophobicity and cytokine release while facilitating liver regeneration. 48 Given this evidence, it raises the question of whether changes in TGR5 expression within bile duct epithelial cells (BECs) correlate with the development of cholestatic diseases. Reich et al 49 found that patients diagnosed with PSC and Abcb4 −/− mice (which serve as a common model for sclerosing cholangitis) exhibited decreased expression of TGR5 in BECs. This reduction was noted early in the disease trajectory, showed specificity to cell types, and was particular to PSC as well as Abcb4 −/− BECs and the extrahepatic bile ducts of Abcb4 −/− mice; it was not encountered in other hepatic conditions such as PBC, nonalcoholic steatohepatitis, drug-induced liver injury, or viral hepatitis. Additional studies have shown that the reduction of TGR5 correlates with intensified damage in BECs. Mice lacking TGR5 demonstrate greater bile duct injury, while enhancing TGR5 expression in Abcb4 −/− models can mitigate PSC and enhance BEC activation along with the inflammatory phenotype.

#### Lipopolysaccharide

Endogenous LPS have been recognized as a contributing factor in the exacerbation of liver injury across various models. Under typical physiological circumstances, the intestinal barrier effectively inhibits LPS from penetrating the bloodstream. Nevertheless, factors such as systemic inflammation, intestinal dysbiosis, or disruption of TJs compromise this barrier's integrity, increasing permeability and allowing LPS to enter circulation. Once in the bloodstream, LPS interacts with TLR4 through CD 14 to trigger the host immune response, which is a crucial mediator of both adaptive and innate immune reactions to LPS. This activation stimulates the release of numerous inflammatory mediators, including TNF-α, as well as the production of granulocyte–macrophage colony-stimulating factor, ultimately resulting in irreversible liver damage. 50 51 Significant evidence underscores the importance of TLR4 signaling, particularly the TLR4/NF-κB pathway, in various liver pathologies, making this axis a valuable target for numerous pharmacological interventions aimed at mitigating liver damage. 52 53 54 In addition, previous studies have indicated that both LPS and soluble CD14 levels are elevated in AILDs, with soluble CD14 being linked to a poorer prognosis. 55 Furthermore, research by Zhang et al 56 revealed that the stimulator of interferon genes (STING) signaling pathway in Kupffer cells plays a role in LPS-induced liver injury. Their study demonstrated that LPS stimulation increased mitochondrial reactive oxygen species (ROS) production dependent on dynamin-related protein 1, which facilitated the release of mtDNA into the cytoplasm and subsequent activation of STING signaling in Kupffer cells. Additionally, their experiments confirmed that the absence of STING afforded protection to liver function, reduced the systemic inflammatory response, and decreased mortality in LPS-treated mice, while the administration of a STING agonist yielded the opposite outcome. By modulating the activation of STING, it is possible to balance the immune response, reduce inflammation, and promote tissue repair. However, further research is needed to clarify the precise mechanisms of STING signaling in AILDs and to develop targeted therapies that can selectively modulate its activity without exacerbating liver injury. The integration of STING regulation with other immunoregulatory pathways, such as the IL-33/ST2 axis 57 and autophagy, 58 may provide a comprehensive approach for treating AILDs and improving patient prognosis.

In recent years, research have indicated that neutrophils represent a significant target for intestinal-derived LPS-induced liver damage. Liu et al 59 attempted to reveal one of the possible mechanisms in their study. They found that in patients with chronic alcoholism, intestinal bacteria and LPS can stimulate the formation of neutrophil extracellular traps (related to tumor immunity in hepatocellular carcinoma [HCC]) through the TLR4 pathway to promote alcoholic liver fibrosis and play a significant role in the development of alcoholic liver cirrhosis and subsequent HCC.

## Clinical and Laboratory Evidence for the Association between the Gut Microbiota and Autoimmune Liver Diseases

### Autoimmune Hepatitis

The current diagnosis of AIH relies on the detection of circulating autoantibodies, increased levels of IgG and gamma globulins, and specific histological alterations. The standard initial treatment approach is the administration of corticosteroids, either alone or in combination with azathioprine. Nevertheless, patients who either do not receive a prompt diagnosis or fail to respond to first-line therapies often experience a notable rise in liver-related morbidity and mortality. 60 Recent studies have investigated the role of gut microbiota in the pathogenesis of AIH, offering new prospects for its early diagnosis and treatment. In their earlier work, Yuksel et al 61 created an innovative AIH mouse model utilizing HLA-DR3 transgenic mice in a nonobese diabetic mouse background, where intestinal dysbiosis was observed in cases of experimental AIH. This investigation revealed an intensified Th1 immune response alongside a reduced frequency of regulatory T cells in the livers of immunized mice. Additionally, research conducted by Manfredo Vieira et al 62 identified a pathogenic bacterium, *E. gallinarum* , within the gut flora. When the intestinal barrier is breached, these bacteria may move into the systemic circulation of hosts susceptible to autoimmune conditions, potentially contributing to the development of autoimmunity. It is conceivable that these translocated bacteria not only affect the differentiation of helper T cells but may also exert direct effects on colonized tissues, including the liver. *Enterococcus gallinarum* has been detected in liver biopsies from patients diagnosed with AIH and cirrhosis, conditions characterized by significant intestinal barrier damage. The translocation of *E. gallinarum* stimulates the activation of the AhR signaling pathway in the liver, subsequently leading to autoimmune liver injury. This process occurs through the induction of pathogenic TH17 cells, the generation of RNA and double-stranded DNA autoantibodies, as well as an inflammatory response.

### Primary Biliary Cholangitis

PBC is marked by nonsuppurative granulomatous and lymphocytic inflammation affecting the small bile ducts within the liver. The challenge of early diagnosis arises from the initial symptoms being masked by PBC and the absence of specific biomarkers. Numerous studies focusing on the compositional analysis of gut microbiota indicate that changes in the community composition of gut microbiota correlate with the progression of PBC. Lv et al 63 employed techniques such as 16S rRNA gene metagenomic sequencing, ultra-performance liquid chromatography-tandem mass spectrometry for small molecule detection, and liquid chip assays for serum cytokines to investigate alterations in the gut microbiota of early-stage PBC patients who did not have other significant health issues. Their findings revealed that, in comparison to healthy subjects, PBC patients exhibited a reduced relative abundance of potentially beneficial bacteria (such as *Acidobacteria* , *Lachnobacterium* sp., and *Bacteroides eggerthii* ), while there was an increase in the presence of pathogenic bacteria (including *Actinobacillus pleuropneumoniae* , *Klebsiella* , *Neisseria* , etc.). Furthermore, they noted that most of the modified intestinal bacteria were linked to liver damage indicators, serum inflammatory cytokines, and metabolic irregularities. Importantly, alterations in the metabolite profiles of blood, urine, and feces were observed in PBC patients, suggesting that changes in intestinal bacteria could indirectly affect metabolism and cholestasis. In a study conducted by Tang et al, 64 a comparative analysis was performed on fecal samples from 79 untreated PBC patients and 114 healthy controls. This research revealed a decrease in the populations of *Bacteroides* , *Faecalibacterium* , *Sutterella* , and *Oscillospira* spp., alongside an increase in *Veillonella* , *Clostridium* , *Lactobacillus* , *Streptococcus* , *Pseudomonas* , *Klebsiella* , and an unidentified genus within the Enterobacteriaceae family ( *Enterobacteriaceae* ) among PBC patients.

The treatment of PBC has traditionally relied on therapies centered around BAs. In a recent study, Li et al 65 demonstrated that the administration of colestipol resulted in a significant decrease in BA levels in the bloodstream, altered the composition of BAs, and lowered the hydrophobic index of these acids. Furthermore, the intervention brought about changes in the intestinal microbiota, notably an increase in bacteria that produce SCFAs among patients who showed a more favorable response to the treatment, alongside a rise in the levels of conditionally pathogenic *K. pneumoniae* in those with poorer responses. These microbiota changes correlated with enhancements in the clinical symptoms of the patients, indicating that BA sequestrants may benefit individuals with PBC by influencing the intestinal microbiome and its metabolic products. Collectively, these findings underscore the potential significance of intestinal microorganisms in both the diagnosis and management of PBC.

### Primary Sclerosing Cholangitis

The long-term prognosis for PSC patients is unfavorable, with up to 40% of patients requiring liver transplantation in the later stages and 20 to 30% developing bile duct cancer. 66 Additionally, there is a considerable risk of developing colorectal cancer. 67 Although ursodeoxycholic acid (UDCA) has been extensively utilized in the treatment of chronic cholestatic liver diseases, with demonstrated efficacy in improving biochemical markers of cholestasis, its ability to prevent the progression of PSC remains limited in most patients. Most patients with PSC also have inflammatory bowel disease (IBD), which makes PSC a model disease for studying the gut–liver axis, suggesting that intestinal microorganisms may contribute to the occurrence of PSC. 68 In a study conducted by Tedesco et al, 69 a comparison was made between a multidrug resistance gene 2 knockout (Mdr2 −/− ) mouse model and an FVB/NJ mouse model. The results demonstrated that the Mdr2 −/− mice exhibited increased intestinal permeability, which resulted in the translocation of intestinal flora to the liver, particularly *Lactobacillus gasseri* . Furthermore, the researchers discovered that γδ T cells extracted from the livers of patients with PSC were capable of producing IL-17, whereas cells from hepatitis C patients were not. This indicates that the activation of γδ TCR+ cells in the liver and the production of IL-17 mediated by translocated *L. gasseri* may be a contributing factor in the development of cholestatic liver diseases such as PSC. At present, researchers are directing their attention to the intestinal fungal community. Lemoinne et al 70 discovered that the intestinal fungal flora of PSC patients is dysbiotic, exhibiting a relative increase in biodiversity and alterations in community composition. Additionally, an increase in the abundance of *Exophiala* spp. and a decrease in the abundance of Saccharomyces cerevisiae were observed. *Exophiala* spp. has been demonstrated to be capable of causing infections in immunocompromised hosts, whereas *S. cerevisiae* has anti-inflammatory properties and has been shown to reduce the recurrence rate in patients with IBD. 71 Based on these findings, Rühlemann et al 72 observed an increased abundance of *Candida* and *Clostridium butyricum* in German PSC patients, whereas *Exophiala* spp. was not detected in the German samples. Further research is necessary to ascertain if these inconsistencies stem from variations in methodology, including the choice of primer sets, tools for data analysis, and the depth of sampling. It can be concluded that individuals with PSC and biliary candidiasis experience more severe cholangitis, alongside elevated levels of C-reactive protein and serum bilirubin, in contrast to those without a Candida infection. 22 Moreover, biliary candidiasis is linked to decreased survival rates among patients suffering from PSC. 73

### IgG4-Related Hepatobiliary Diseases

IgG4-related hepatobiliary disorders are immune-mediated conditions that are closely associated with IgG4-RD. This group primarily encompasses IgG4-related sclerosing cholangitis (IgG4-SC), IgG4-related hepatopathy, and IgG4-related cholangitis. The subtle onset, gradual progression, and infrequent emergence of severe clinical symptoms or acute organ failure in these conditions often result in many patients having some level of irreversible organ dysfunction by the time they receive a diagnosis. 74

While extensive research has been conducted on intestinal dysbiosis in AIH, PBC, and PSC, the involvement of gut microbiota in IgG4-related hepatobiliary diseases remains uncertain. Liu et al 75 compared the stool composition of patients diagnosed with IgG4-SC to that of those with PSC and healthy controls. Their integrated multiomics analyses, incorporating 16S rRNA gene amplicon sequencing along with untargeted metabolomics, revealed key characteristics of the fecal microbiome in IgG4-SC, including reduced intraspecific diversity and altered microbiome structure. These differences were significant when comparing IgG4-SC patients to both PSC and healthy controls. Moreover, the research identified 58 metabolites exhibiting varying abundances in IgG4-SC in relation to healthy controls. Notably, specific intestinal microbial metabolites, particularly succinic acid and L-palmitoylcarnitine, showed significant elevation in patients with IgG4-SC. Prior investigations have indicated that alterations in succinic acid signaling are linked to the activation and functionality of immune cells, 76 whereas L-palmitoylcarnitine is also believed to influence inflammatory activation. 77 Importantly, several studies have indicated that microbiome-derived succinic acid can stimulate type II immune responses, 78 which aligns with the observed type II immunity skewing in IgG4-SC. In conclusion, despite certain limitations of this study (including being a single-center study with a small sample size), it provides evidence suggesting that the alterations in intestinal microbiota and metabolic processes in IgG4-SC may play a role in the disease's inflammatory mechanisms.

## The Potential Value of Gut Microbiota in the Diagnosis and Treatment of Autoimmune Liver Diseases

### The Possibility of Specific Flora as Diagnostic Markers

Accumulating evidence suggests that gut microbiota profiles are associated with a variety of diseases, including HCC, 79 IBD, 80 colorectal cancer, and Parkinson's disease. Similarly, microbial alterations observed in AILDs have been proposed as potential early indicators, although their diagnostic value remains to be validated ( Table 3 ).

**Table 3:** Altered gut microbiota associated with autoimmune liver diseases

Models	Disease	Specific flora	Patients ( *N* )	Controls	References
Human Human Human Human Human Human/Mice Human Human	AIH AIH PBC PBC PBC PSC PSC IgG4-SC	Enrichment of *Veillonella* , *Klebsiella* , *Streptococcus* , and Lactobacillus; Depletion of e.g., *Clostridiales* , *Oscillospira* , *Coprococcus* Depletion of *Bifidobacterium* , *Faecalibacterium* ; Enrichment of *Veillonella* , *Lactobacillus* , *Streptococcus* Enrichment of *Enterobacteriaceae* .g, *Veillonella* , *Klebsiella* ; Depletion of *Faecalibacterium* and *Sutterella* , both increased after UDCA treatment Enrichment of *Lactobacillus* , *Enterococcus* ; Depletion of *Faecalibacterium* , *Anaerostipes* Enrichment of e.g., *Streptococcus* , *Veillonella* , *Haemophilus* Enrichment of *Klebsiella pneumoniae* , *Proteus mirabilis* , *Enterococcus faecium* Enrichment of *Enterococcus* , *Fusobacterium* , *Lactobacillus* ; Depletion of *Faecalibacterium* , *Ruminococcaceae* , *Roseburia* Enrichment of *Streptococcus* ; Depletion of *Blautia* , *Lachnospiraceae* ND3007	119 72 79 76 60 17 PSC GF-mice 23 PSC + UC 11 PSC only 13 IgG4-SC 15 PSC	132 HC 95 HC 99 PBC 81 UC 114 HC 23 HC 60 HC 13 HC 23 UC 22 CD 68 HC 15 HC	81 85 64 86 63 16 88 75

Yiran et al 81 conducted an analysis of the gut microbiota in patients with AIH compared with healthy individuals. The results indicated that, in comparison to the control group, the abundance of several bacterial species was significantly reduced in AIH patients, including *Clostridiales* , RF 39, *Ruminococcaceae* , *Rikenellaceae* , *Oscillospira* , *Parabacteroides* , and *Coprococcus* . Conversely, the gut microbiota of AIH patients exhibited an increased abundance of *Veillonella* , *Klebsiella* , *Streptococcus* , and *Lactobacillus* compared with the control group. Interestingly, earlier studies have also reported that altered abundances of *Veillonella* and *Lactobacillus* in patients with PBC and PSC, suggesting a potential association with disease-related microbial dysbiosis. 82 83 84 Of note, *Veillonella dispar* positively correlated with serum level of AST and liver inflammation was the most strongly disease-associated taxa in this study. Based on the above, they subsequently applied multivariable stepwise logistic regression analysis to determine the most obvious classified flora of AIH and controls, the authors ultimately suggested that a microbial signature comprising *Veillonella* , *Lactobacillus* , *Oscillospira* , and *Clostridiales* exhibited promising discriminatory power for distinguishing AIH patients from controls within their study cohort. However, it is important to emphasize that the findings are based on correlational analysis and do not imply causality. In addition, the only study to date that directly compares different ALIDs was conducted by *Liwinski* et al, 85 they sequenced stool samples from AIH patients and control groups (including healthy controls, PBC controls, and ulcerative colitis (UC) controls). Similarly, they also observed significant changes in the relative abundance of *Veillonella* and *Lactobacillus* , as well as *Bifidobacterium* , and *Faecalibacterium* in the AIH group, where a significant reduction in the number of *Bifidobacterium* was observed in patients, which was associated with the AIH *Bifidobacterium* was significantly reduced in the patients, which was related to the degree of AIH remission.

In the aforementioned study by Lv et al, 63 receiver operating characteristic curve analysis suggested that *Streptococcus* sp. and *Veillonella* sp. may may have potential discriminatory value in differentiating patients with PBC from healthy controls. Furthermore, Tang et al 64 reported that several genera, including *Enterobacteriaceae* (unclassified genus), *Klebsiella* , *Veillonella* , and *Streptococcus* , were significantly enriched in PBC patients, with *Enterobacteriaceae* showing the strongest association. In contrast, *Faecalibacterium* —a well-known butyrate-producing genus with anti-inflammatory properties—was significantly reduced in PBC compared with healthy controls. These findings suggest a microbial imbalance in PBC, characterized by an overrepresentation of potentially pathogenic bacteria and depletion of beneficial taxa such as *Faecalibacterium* , although causality has yet to be established. Building on these foundation, *Furukawa* et al 86 analyzed fecal samples from 76 Japanese patients with PBC, of whom 73 had been treated with UDCA for more than 1 year. Using 16S rRNA gene sequencing, they compared the gut microbiota profiles of PBC patients with those of healthy controls. The study revealed that, compared with healthy individuals, the relative abundance of the order *Clostridiales* , including butyrate-producing genera such as *Faecalibacterium* , *Roseburia* , and *Anaerostipes* —was significantly reduced in PBC patients. In contrast, members of the order *Lactobacillales* , including *Lactobacillus* , *Enterococcus* , and *Streptococcus* , were markedly enriched. Notably, the abundance of *Faecalibacterium* was significantly lower in UDCA nonresponders compared with responders, as defined by the Nara criteria, suggesting a potential association between reduced *Faecalibacterium* levels and poor treatment response. Additionally, Zhang et al 87 conducted a two-sample bidirectional Mendelian randomization study to assess the causal relationship between gut microbiota composition and PBC. The analysis revealed that a higher abundance of certain taxa, such as Ruminococcaceae, Peptostreptococcaceae, Christensenellaceae R7 group, Anaerofilum, Ruminococcaceae UCG-013, and Holdemania, was causally associated with a lower risk of PBC. Among these, Ruminococcaceae is known to include butyrate-producing genera, suggesting a potential protective role through maintaining intestinal barrier integrity and reducing systemic immune activation. Conversely, taxa such as Selenomonadales, Bifidobacteriales, Lachnospiraceae UCG-004, Oscillospira, and Eubacterium nodatum group were positively associated with increased PBC risk. These findings highlight specific microbial taxa that may play divergent roles in the pathogenesis of PBC and warrant further mechanistic investigation.

Sabino et al 88 demonstrated that intestinal dysbiosis in patients with PSC is independent of coexisting IBD. After controlling for potential confounders including antibiotic and probiotic use, UDCA treatment, liver cirrhosis, and liver transplantation, the genera *Enterococcus* , *Lactobacillus* , and *Fusobacterium* were significantly enriched in PSC patients regardless of IBD status or treatment. These findings suggest that PSC is characterized by a distinct gut microbial signature that is not secondary to IBD or medication exposure. As previously stated, Nakamoto et al 16 identified an enrichment of *K. pneumoniae* , *P. mirabilis* , and *E. faecium* in the gut microbiota of PSC patients. These bacteria were shown to impair intestinal barrier integrity and induce hepatic Th17 responses in gnotobiotic mouse models, supporting their potential pathogenic role in PSC progression. Among them, *K. pneumoniae* exhibited the strongest capacity to trigger Th17-mediated inflammation. These findings suggest that tracking the abundance of these pathobionts, particularly *K. pneumoniae* , may provide insights into PSC pathogenesis and hold potential value for future diagnostic strategies.

In the study by Liu et al, 75 although a substantial overlap in gut microbial alterations was observed between patients with IgG4-SC and PSC, three taxa exhibited relatively higher specificity in IgG4-SC: enrichment of *Streptococcus* and depletion of *Blautia* and *Lachnospiraceae* _ND3007_group. These distinct microbial signatures may provide potential clues for differentiating IgG4-SC from PSC based on microbiome analysis.

It remains unclear whether the observed reduction in gut microbiota diversity in patients with AILDs is primarily driven by the disease process itself or by therapeutic interventions, such as UDCA, antibiotics, or immunosuppressants. Most existing studies rely on 16S rRNA gene sequencing, which, although useful for community-level profiling, lacks the resolution to distinguish microbial taxa at the species or strain level. To address these limitations, future research should incorporate higher-resolution approaches such as whole-genome shotgun metagenomics, 55 metatranscriptomics, and culture-based techniques, which allow more accurate identification of functionally relevant strains. Additionally, a more comprehensive stratification of disease status is essential. In particular, IBD, commonly coexisting with PSC, represents a major confounding factor in gut microbiome analyses. The influence of IBD activity and its treatments on microbial diversity and metabolic output should be carefully controlled in future studies.

### Application of Probiotics

**Fig. 3:**
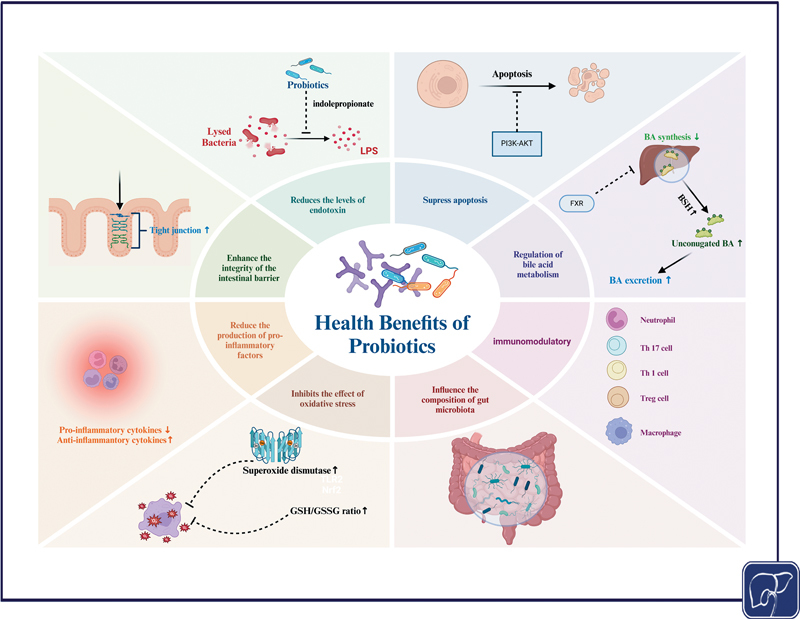
Multifunctional mechanisms of probiotics in promoting host health. *Created in BioRender. ShiHui, W. (2025)* *https://BioRender.com/t81t864* .

The administration of particular probiotics has the potential to restore the equilibrium of the gut microbiota and mitigate the inflammatory response in autoimmune disorders ( Fig. 3 ). For example, supplementation with Bifidobacterium has been demonstrated to balance the Treg/Th17/Th1 ratio, thereby preventing excessive activation of CD4+ lymphocytes. 89 Zhang et al 90 found that *Bifidobacterium animalis* ssp. lactis 420 (B420) can significantly alleviate s100-induced experimental AIH (EAH) by regulating the RIP3 signaling pathway and cytokine profile of hepatic macrophages to inhibit Th17 cell differentiation. B420 also strengthens the intestinal barrier by upregulating TJ proteins. In addition, B420 alters the composition of the mouse gut microbiota, which is characterized by a decrease in *Bacteroides* , *Ruminococcus* , and an increase in *Lactobacillus* , *Alistipes* , and *Rikenella* at the genus level. Ma et al 91 selected 50 patients with active AIH who had not received any drug intervention and established an experimental mouse model of AIH to analyze the effect of prednisone combined with *Lactobacillus* treatment. The study found that compared with patients treated with prednisone alone, those treated with Lactobacillus-prednisone had significantly higher levels of *Bacteroides fragilis* , *Clostridium* , *Clostridium* *leptum* , and *Bifidobacterium* in their stools and a more significant reduction in the relative levels of alanine aminotransferase (ALT), aspartate aminotransferase (AST), smooth muscle antibody, antinuclear antibody, IgG, and so on in the serum of patients. Further studies in the EAH mouse model showed that *Lactobacillus* *reuteri* improved the therapeutic effect of prednisone, and the two may regulate the Tfh response in EAH mice through the TLR4/MyD88/NF-κB pathway. The findings indicate that combining *Lactobacillus reuteri* supplementation with prednisone may offer therapeutic benefits in managing AIH.

Furthermore, Wu et al 92 conducted an animal experiment and a randomized controlled clinical trial to evaluate the effect of *Lactobacillus* *acidophilus* on patients with cholestasis. The results of the animal experiment showed that *L. acidophilus* significantly alleviated liver damage in mice by activating intestinal FXR signaling to inhibit the synthesis of BAs in the liver, increasing unbound BAs by accumulating bile salt hydrolase, and promoting the excretion of BAs. In addition, a randomized controlled clinical trial found that *L. acidophilus* supplementation can promote recovery of liver function in patients, providing another treatment option for cholestatic liver disease. A study of patients with PBC also revealed that probiotic supplementation, including *Lactobacillus* , may assist in reducing symptoms and improving liver function indicators. Moreover, it has been demonstrated that metabolites of probiotics, such as indolepropionate, can mitigate the immune response of the liver by reducing endotoxin levels. 93 *Akkermansia muciniphila* is regarded as a “new generation of probiotics” that exerts a beneficial influence in a multitude of ailments, including diabetes, obesity, cancer, and metabolic syndrome. 94 Xia et al 95 observed in a mouse model of acetaminophen-induced liver injury that *A. muciniphila* alleviated oxidative stress in the liver by regulating the reduced glutathione/oxidized glutathione (GSH/GSSG) balance and enhancing superoxide dismutase activity. Additionally, it has been demonstrated to diminish the generation of proinflammatory cytokines, inhibit the infiltration of macrophages and neutrophils in the liver, and mitigate hepatocyte apoptosis by activating the phosphoinositide 3-kinase (PI3K)/Akt signaling pathway. Nevertheless, the majority of the research is based on animal models, and it remains to be seen whether the same effect can be observed in liver damage induced by other factors and whether the results can be replicated in humans. Further animal experiments and clinical studies are necessary to verify these hypotheses. Moreover, the utilization of its regulation of the gut microbiota and its metabolites and the enhancement of intestinal barrier integrity to treat AILDs-related increased intestinal permeability represents a further potential avenue of investigation.

As discussed in earlier sections, several genera traditionally regarded as probiotics, such as *Lactobacillus* , *Bifidobacterium* , and *Enterococcus* , have been reported to be enriched in patients with AILDs. This raises potential concerns about their indiscriminate use in therapeutic settings. Although specific probiotic strains have demonstrated immunomodulatory and hepatoprotective effects in animal models or pilot clinical trials, their applicability to patients with preexisting dysbiosis requires careful consideration. Notably, emerging evidence also points to potential risks of probiotic-related bacteremia, 96 and that probiotic intake may affect the evolution of resident intestinal microorganisms, potentially altering their genomic content, metabolic functions, or resistance patterns over time. 97 Moreover, emerging evidence from the broader probiotic safety literature indicates that probiotic strains can harbor antibiotic resistance genes and act as reservoirs for HGT. 98 99 100 Common probiotic species such as *Lactobacillus* and *Bifidobacterium* have been found to carry mobile resistance determinants like tet, erm, and van genes. Some of these genes are plasmid-borne and have been shown to be transferable to pathogenic or commensal bacteria in both in vitro and in vivo studies. These findings underscore the need for strain-level genomic screening and resistome profiling prior to the use of probiotics as adjunctive therapy in AILDs, particularly in patients with compromised gut barriers or immunological dysfunction.

Probiotics can reduce the level of endotoxin in the body by inhibiting the production of endotoxin by lysogenic bacteria, while inhibiting apoptosis through the PI3K–AKT pathway. In BA metabolism, probiotics can reduce BA synthesis through the FXR pathway, increase BSH activity to increase unconjugated BAs and promote their excretion. Furthermore, probiotics display anti-inflammatory properties by enhancing the expression of TJ proteins, strengthening the intestinal barrier's integrity, decreasing the synthesis of proinflammatory substances, and elevating the concentrations of anti-inflammatory factors. Probiotics also have immunomodulatory effects, affecting the activity of various immune cells, including neutrophils, Th17 cells, Th1 cells, Treg cells, and macrophages. Probiotics can regulate the composition of the intestinal microbiota, maintain intestinal health, and at the same time inhibit the harmful effects of oxidative stress by increasing the expression of superoxide dismutase and increasing the GSH/GSSG ratio ( Fig. 2 ).

### Fecal Microbiota Transplantation

Transferring fecal microbiota from a healthy donor into a patient's intestine is becoming a promising therapy for autoimmune diseases linked to microbial factors. Fecal microbiota transplantation (FMT) is most commonly associated with the treatment of recurrent *Clostridioides difficile* infection. 101 Furthermore, studies have indicated that, in addition to gastrointestinal disorders such as IBD and *Helicobacter pylori* infection, 102 seemingly nongastrointestinal disorders may also serve as potential therapeutic targets for FMT, for example non-alcoholic steatohepatitis, hepatic encephalopathy, depression, etc. 103 Given the above, restoring the gut microbiota through FMT to improve the condition of patients with PSC seems like a good idea. A pilot clinical trial conducted by Allegretti et al 104 investigated the effects of FMT in patients with PSC and concurrent IBD. Of the 14 enrolled participants, 10 completed the FMT protocol, which consisted of six weekly administrations. The study observed a significant increase in gut microbial α-diversity following treatment. Additionally, 3 out of 10 patients (30%) experienced a ≥50% reduction in serum alkaline phosphatase levels, indicating a biochemical response. Although reductions in serum transaminases (ALT and AST) were reported in some individuals, detailed stratified data were not provided. Importantly, no serious adverse events related to FMT were observed during the study. Ma et al 105 investigated the therapeutic effects of FMT in a mouse model of EAH induced by hepatic antigen S100. The study demonstrated that FMT significantly reduced serum levels of ALT, AST, and total bilirubin, and alleviated histological liver inflammation. Mechanistically, FMT reversed the elevation of proinflammatory cytokine IL-21 and promoted the expression of anti-inflammatory cytokines IL-10 and TGF-β. Additionally, FMT restored the balance between follicular helper T (Tfh) cells and follicular regulatory T (Tfr) cells, potentially via downregulation of the TLR/MyD88 signaling pathway.

Although FMT shows promising immunomodulatory effects in preclinical studies of ALIDs, several potential risks should be considered. These include donor-derived pathogen transmission, unpredictable immune responses due to host–microbiota interactions, immunological imbalance from unintended microbial shifts, and the lack of standardized protocols for donor screening and microbial composition. The evidence to date remains limited, especially for PBC and IgG4-SC, and further large-scale, controlled clinical trials are warranted to validate its safety and efficacy in AILDs.

### Intervention with Microbial Metabolites

Myeloid-derived suppressor cells (MDSCs) have been shown to have potent immunosuppressive functions by upregulating inducible nitric oxide synthase, ROS, arginase 1, and prostaglandin E2. A 2018 study by Zhang et al 106 showed that MDSC accumulation in PBC patients was negatively correlated with disease severity. On this basis, Wang et al 107 recently found that gut microbial butyrate promotes the differentiation and function of MDSCs by inhibiting HDAC3, enhancing their immunosuppressive capacity and thus ameliorating the condition of PBC, providing a new potential target for the treatment of PBC.

Recent studies have highlighted the potential of combining Quercetin and *A. muciniphila* as a therapeutic strategy for immune-related liver diseases. Juárez-Fernández et al 108 divided 21-day-old rats into a control group and a high-fat diet group and fed them a control diet and a high-fat diet for 6 weeks. Then, quercetin and/or *A.* *muciniphila* were added to the control group diet with/without quercetin for 3 weeks. The results showed that *A.* *muciniphila* and quercetin could significantly increase the level of total BAs in plasma, among which the concentration of primary BAs increased the most. In addition, the synbiotic combination of *A.* *muciniphila* and quercetin increased the ratio of unconjugated to conjugated BAs. Specifically, plasma concentrations of all unconjugated primary BA species increased after supplementation with this synbiotic, with the most significant increases in cholic acid (CA), β-murucic acid (βMCA), and α-murucic acid (αMCA). CA has been shown to be negatively correlated with TLR2 expression, which may be related to protection against inflammation. In addition, the increase in MCA levels means that the toxicity and hydrophobicity of the BA pool is reduced, creating a healthier hydrophilic BA pool. Beyond BA modulation, quercetin also exerts anti-inflammatory effects through Nrf2/HO-1 pathway activation and NF-κB suppression. 109 Simultaneously, *A.* *muciniphila* has been shown to enhance intestinal barrier integrity and modulate immune responses by shaping γδT17 cell activity and macrophage polarization. 110 More strikingly, a recent study revealed that the combination of *A.* *muciniphila* and inosine restored the Treg/Th17/Th1 balance and upregulated key immunoregulatory markers—CD39, CD73, and the adenosine A2A receptor—within the gut–liver axis, offering protection against alcohol-induced liver injury. 111 However, the current evidence supporting the use of *A.* *muciniphila* and quercetin in AILDs remains preliminary and largely extrapolated from non-AILD models. Future studies should incorporate AILD-specific animal models and patient-derived data to validate these effects. Moreover, attention should be given to the complex immunopathology of AILDs, potential strain- and dose-specific effects, and safety profiles before clinical application. Integration of multiomics approaches and personalized intervention strategies may further enhance the translational potential of this synbiotic combination.

## Other Therapeutic Strategies Based on Intestinal Microecological Modulation

### Antibiotics

It has been demonstrated that the administration of antibiotics to eradicate *E. faecium* can mitigate the progression of extraintestinal autoimmune diseases. 62 A pilot clinical trial has also indicated a potential therapeutic effect of metronidazole and vancomycin in the treatment of PSC. 112 Furthermore, a systematic review and meta-analysis were performed by Shah et al 113 explore the efficacy of antibiotics in treating PSC, both with and without IBD. Their results indicate that vancomycin could be the most promising antimicrobial option for managing PSC. The researchers aim to utilize more selective antibiotics to avoid targeting other symbiotic flora and to develop new mechanisms of action to prevent antibiotic resistance, with the objective of achieving long-term stable medication. However, the optimal antibiotic drug, dosage, regimen, and potential long-term side effects remain largely unknown. Even if targeted eradication of pathogenic bacteria is achieved, it is possible that unintended consequences may arise through indirect changes to the microbial ecological balance.

### Monocytes

In a study conducted by Kunzmann et al, 114 it was observed that, in comparison to patients diagnosed with PBC and healthy individuals, patients with PSC had a noticeably greater quantity of CD4+ T cells that produced IL-17A in the peripheral blood. Additionally, patients with PSC-associated cirrhosis exhibited augmented numbers of CD14hiCD16int and CD14loCD16hi monocyte–macrophages in the vicinity of bile ducts within the liver, when compared with individuals with alcoholic cirrhosis. The study revealed that monocytes from patients with PSC exhibited heightened production of IL-1β and IL-6 following stimulation with *C. albicans* and *Enterococcus faecalis* (which may translocate in the context of increased intestinal barrier permeability). These two cytokines are known to drive the differentiation of Th17 cells. The results indicate that monocytes may serve as a functional conduit between proinflammatory microbiota and T cells and may contribute to the pathogenesis of PSC. Accordingly, future research should consider the potential of monocytes as a therapeutic target for PSC.

### Vaccination

The feasibility of vaccination against pathogenic bacteria for microbiologically induced immune diseases is still under investigation. This is due to the fact that the same pathogenic bacteria may play a beneficial role in normal or other disease conditions, as well as easily remove or cause ecological disorders in the microbiota. However, intramuscular vaccination against the pathogenic bacterium *Enterococcus avium* in animal models has been demonstrated to be safe and effective in preventing translocation to internal organs and systemic autoimmunity. 62

## Conclusion

The complex interplay between the gut microbiota and the host immune system plays a pivotal role in the pathogenesis of AILDs by modulating intestinal barrier integrity, immune homeostasis, and the production of bioactive metabolites. Despite substantial advances in delineating the association between intestinal microecology and AILDs, current research remains limited by several constraints. The high interindividual variability and complexity of the gut microbial ecosystem present significant challenges for its therapeutic exploitation. Moreover, most existing studies concentrate on individual microbial taxa or specific metabolites, offering only a fragmented view of the dynamic equilibrium between the microbiome and host immunity.

Further investigations are needed to elucidate the specific mechanisms through which the gut microbiota contributes to the onset and progression of distinct AILD subtypes and to determine whether these microbial alterations are causal or consequential. Translational strategies, such as probiotic and prebiotic supplementation, FMT, and microbial metabolite modulation have shown preliminary potential in both experimental and clinical settings. However, their clinical implementation is still hindered by uncertainties regarding safety, efficacy, and durability of effect. Rigorous, large-scale clinical trials are essential to validate these approaches and to optimize microbiota-based interventions in the management of AILDs.

## Notes

Lay Summary Autoimmune liver diseases (AILDs), including autoimmune hepatitis, primary biliary cholangitis, and primary sclerosing cholangitis, are chronic conditions caused by the immune system attacking the liver. Recent studies have revealed that the gut microbiota, the vast community of microbes living in the intestine, plays a critical role in shaping liver immunity and disease progression. These microbes influence liver health by affecting intestinal barrier integrity, modulating immune responses, and producing bioactive metabolites. Moreover, disturbances in gut microbial composition have been associated with the onset and severity of AILDs. This review summarizes the latest findings on how gut–liver communication contributes to autoimmunity and explores emerging microbiota-based therapies, such as probiotics, prebiotics, and fecal microbiota transplantation. Understanding the gut–liver axis may pave the way for novel diagnostic and therapeutic approaches in AILDs.
